# The enhancement of photosynthetic performance, water use efficiency and potato yield under elevated CO_2_ is cultivar dependent

**DOI:** 10.3389/fpls.2023.1287825

**Published:** 2023-11-16

**Authors:** Keshav Dahal, Matthew A. Milne, Taylor Gervais

**Affiliations:** Fredericton Research and Development Centre, Agriculture and Agri-Food Canada, Fredericton, NB, Canada

**Keywords:** potato cultivars, elevated CO_2_, photosynthesis, water use efficiency, growth, tuber yield, tuber number

## Abstract

As a fourth major food crop, potato could fulfill the nutritional demand of the growing population. Understanding how potato plants respond to predicted increase in atmospheric CO_2_ at the physiological, biochemical and molecular level is therefore important to improve potato productivity. Thus, the main objectives of the present study are to investigate the effects of elevated CO_2_ on the photosynthetic performance, water use efficiency and tuber yield of various commercial potato cultivars combined with biochemical and molecular analyses. We grew five potato cultivars (AC Novachip, Atlantic, Kennebec, Russet Burbank and Shepody) at either ambient CO_2_ (400 μmol CO_2_ mol^−1^) or elevated (750 μmol CO_2_ mol^−1^) CO_2_. Compared to ambient CO_2_-grown counterparts, elevated CO_2_-grown Russet Burbank and Shepody exhibited a significant increase in tuber yield of 107% and 49% respectively, whereas AC Novachip, Atlantic and Kennebec exhibited a 16%, 6% and 44% increment respectively. These differences in CO_2_-enhancement of tuber yield across the cultivars were mainly associated with the differences in CO_2_-stimulation of rates of photosynthesis. For instance, elevated CO_2_ significantly stimulated the rates of gross photosynthesis for AC Novachip (30%), Russet Burbank (41%) and Shepody (28%) but had minimal effects for Atlantic and Kennebec when measured at growth light. Elevated CO_2_ significantly increased the total tuber number for Atlantic (40%) and Shepody (83%) but had insignificant effects for other cultivars. Average tuber size increased for AC Novachip (16%), Kennebec (30%) and Russet Burbank (80%), but decreased for Atlantic (25%) and Shepody (19%) under elevated versus ambient CO_2_ conditions. Although elevated CO_2_ minimally decreased stomatal conductance (6–22%) and transpiration rates (2–36%), instantaneous water use efficiency increased by up to 79% in all cultivars suggesting that enhanced water use efficiency was mainly associated with increased photosynthesis at elevated CO_2_. The effects of elevated CO_2_ on electron transport rates, non-photochemical quenching, excitation pressure, and leaf chlorophyll and protein content varied across the cultivars. We did not observe any significant differences in plant growth and morphology in elevated versus ambient CO_2_-grown plants. Taken all together, we conclude that the CO_2_-stimulation of photosynthetic performance, water use efficiency and tuber yield of potatoes is cultivar dependent.

## Introduction

Atmospheric CO_2_ concentration is predicted to continue to rise from current ambient CO_2_ concentration of about 400 μmol CO_2_ mol^−1^ (NASA, 2023) to approx. 700 μmol CO_2_ mol^−1^, by the end of the 21^st^ century (IPCC, 2007). Such increase in atmospheric CO_2_ concentration may profoundly affect plant growth, photosynthetic performance and crop productivity worldwide. The anticipated increase in atmospheric CO_2_ concentration is expected to have positive effects on most C_3_ species particularly through stimulation of rates of photosynthesis and water use efficiency (WUE), which may consequently enhance plant biomass and yield (Ainsworth and Long, 2005; Ainsworth and Rogers, 2007; Xu et al., 2013; Dahal et al., 2014; van der Kooi et al., 2016; Dahal and Vanlerberghe, 2018). The extent of CO_2_-stimulation of rates of photosynthesis and plant biomass is, however, dependent on the temperature, water status, nutrient status and growth facilities (Leakey et al., 2009). Previous studies have suggested that the increase in atmospheric CO_2_ concentration by double the current CO_2_ level is predicted to stimulate growth and yield of most C_3_ crop species by up to 36% (Kimball and Idso, 1983; Cure and Acock, 1986) mainly through increase in carbon assimilation and WUE, and decrease in photorespiration (Ainsworth and Long, 2005; Ainsworth and Rogers, 2007; Dahal and Vanlerberghe, 2018; Wujeska-Klause et al., 2019). Growth of plants under elevated CO_2_ is also believed to suppress stomatal conductance leading to reduced transpiration water loss and consequently improved WUE (Ainsworth and Rogers, 2007; Leakey et al., 2009; Katul et al., 2010; Lammertsma et al., 2011; Ji et al., 2015; Engineer et al., 2016; Cao et al., 2022).

C_3_ plants stimulate rates of photosynthesis following shifts from ambient to an elevated CO_2_ (Cheng et al., 1998; Long et al., 2004; Ainsworth and Rogers, 2007; Dahal et al., 2012; Kaiser et al., 2017; Zheng et al., 2022). This CO_2_− induced stimulation of rates of carbon assimilation in C_3_ plants following a shift to elevated CO_2_ is attributed to two reasons: First, at current atmospheric CO_2_ concentration of about 400 μmol CO_2_ mol^−1^, Ribulose-1,5-bisphosphate carboxylase/oxygenase (Rubisco), a key photosynthetic enzyme that assimilate CO_2_ to carbohydrates, is CO_2_ substrate-limited. This is because the Michaelis constant K_m_ (CO_2_) for Rubisco is close to the current ambient CO_2_ concentration of approximately 400 μmol CO_2_ mol^−1^ (Long et al., 2004; Tcherkez et al., 2006). Thus, an immediate enhancement of Rubisco carboxylation velocity can be expected due to increased availability of CO_2_ substrate for Rubisco following a shift to elevated CO_2_. Second, because CO_2_ competitively inhibits the Rubisco oxygenation of RUBP, elevated CO_2_ will lead to reduction in photorespiratory carbon loss (Long et al., 2004; Ehlers et al., 2015; Wujeska-Klause et al., 2019). As a result, as opposed to Rubisco oxygenation reaction, the assimilatory power, NADPH and ATP, is preferentially utilized in Rubisco carboxylation reaction to assimilate CO_2_. Conversely, long-term growth and development of C_3_ plants at elevated CO_2_ may lead to feedback inhibition of initial photosynthetic stimulation gained following short-term shift (Paul and Foyer, 2001). This may trigger several potential downstream effects on plant growth, development, and metabolism (MacNeill et al., 2017). The feedback inhibition of photosynthesis under long-term growth at elevated CO_2_ is attributed in part to P_i_ regeneration limitation in the chloroplast leading to ATP depletion (Stitt and Quick, 1989; Pammenter et al., 1993). Additionally, prior studies have revealed that long-term growth at elevated CO_2_ results in the reduction of the transcript levels for major photosynthetic genes, and subsequent amount and activities of corresponding proteins (Kramer, 1981; Stitt and Quick, 1989; Arp, 1991; Drake et al., 1997; Moore et al., 1999; Byeon et al., 2021).

Potato is one of the principal food crops after wheat, corn and rice feeding over a billion people worldwide (FAOSTAT, 2021). It is cultivated in over 100 countries, and the total potato production in 2021 was estimated to be 376 million tons globally. Potatoes are rich source of essential nutrients such as carbohydrates, dietary fiber, protein, vitamins, antioxidants and minerals (Beals, 2019). Improving potato crop productivity under projected increasing CO_2_ concentrations may therefore contribute to secure the global nutritional demand of the rising population (Birch et al., 2012), which is predicted to reach to about 9.7 billion by 2050 (United Nations et al., 2022). Potatoes have also been considered as a candidate food crop to support future space life because of its high yield potential, balanced nutritional values and high harvest index (Wheeler et al., 2019).

Experiments on effects of increasing CO_2_ concentrations on the physiological, morphological and growth characteristics of potato plants have been performed under various growth settings including growth chambers (Cao et al., 1994; Mackowiak and Wheeler, 1996; Wheeler and Tibbitts, 1997; Donnelly et al., 2001; Fleisher et al., 2008; Kaminski et al., 2014; Lee et al., 2020; Chen and Setter, 2021; Igarashi et al., 2021), greenhouses (Goudriaan and de Ruiter, 1983; Chen and Setter, 2021), open top chambers (Sicher and Bunce, 1999; Donnelly et al., 2001; Lawson et al., 2001; Sicher and Bunce, 2001; Finnan et al., 2002; Katny et al., 2005; Aien et al., 2014; Fleisher et al., 2021) and under free air carbon dioxide enrichment (FACE) systems (Miglietta et al., 1997; Miglietta et al., 1998; Finnan et al., 2005; Fleisher et al., 2021). Although experimental results have varied widely under various growth settings, an increase in tuber yield, photosynthetic performance, and water use efficiency has been generally observed upon growth of potato plants at elevated CO_2_.

In a growth chamber experiment, growth of potato plants at elevated CO_2_ stimulated rates of photosynthesis by 62% at 700 μmol CO_2_ mol^−1^ and by 43% at 1000 μmol CO_2_ mol^−1^ relative to those rates at ambient CO_2_ (Kaminski et al., 2014). Stomatal conductance and transpiration rates were suppressed by 21% and 19% at 700 μmol CO_2_ mol^−1^, and by 43% and 40% respectively at 1000 μmol CO_2_ mol^−1^ in comparison to those rates at ambient CO_2_ (Kaminski et al., 2014). This was reflected in increased WUE of 89% at 700 μmol CO_2_ mol^−1^, and of 147% at 1000 μmol CO_2_ mol^−1^, compared to at ambient CO_2_ (Kaminski et al., 2014). Wheeler et al. (1991) reported an increase in tuber yield and plant dry weight upon growth at elevated CO_2_, the extent of increment, however varied with growth irradiance and photoperiod. For instance, growth of potatoes at elevated CO_2_ increased tuber yield by 39% and total plant dry weight by 34% under 12h photoperiod at 400 μmol m^−-2^ s^−1^ irradiance. However, this stimulation was only 27% and 19% under 12h photoperiod at 800 μmol m^−2^ s^−1^. Under 24h photoperiod, both total tuber yield and plant dry weight increased by 9% at 400 μmol m^−2^ s^−1^ but decreased both by 9% at 800 μmol m^−2^ s^−1^ relative to at ambient CO_2_ (Wheeler et al., 1991). In another study, Wheeler et al. (2019) observed that compared to at 400 μmol CO_2_ mol^−1^, growth of potato plants at elevated CO_2_ of 1000 μmol CO_2_ mol^−1^ enhanced the rates of net photosynthesis by 36% and 27% at 400 and 800 μmol m^−2^ s^−1^ respectively under 12h photoperiod. However, under 24h photoperiod, these rates were decreased by 11% and 20% at 400 and 800 μmol m^−2^ s^−1^ respectively at elevated CO_2_ relative to at ambient CO_2_. Wheeler et al. (2019) revealed that the increase in tuber yield under elevated CO_2_ was accounted for by increase in the rates of photosynthesis and reduction in stomatal conductance.

In an open top chamber study, the potato cultivar ‘Bintje’ exhibited about 40% increase in tuber yield upon growth at either 550 or 680 μmol CO_2_ mol^−1^ of elevated CO_2_ in comparison to at ambient CO_2_ (Donnelly et al., 2001). This stimulation was mainly accounted for by increase in average tuber size with tuber number having minimal effect. However, in a similar study, Lawson et al. (2001) reported that although elevated CO_2_ stimulated above‐ground biomass and tuber dry weight of cv. Bintje during the early stages of the growth season, this stimulation was not observed later at maturity. The authors reported an increase in tuber numbers only, but not tuber yield at maturity under elevated versus ambient CO_2_ growth conditions (Lawson et al., 2001). Similarly, Aien et al. (2014) observed up to 24% increase in leaf photosynthesis, 40% in tuber yield and 36% increase in plant biomass at 570 μmol CO_2_ mol^−1^ as compared to at 385 μmol CO_2_ mol^−1^.

The effects of elevated CO_2_ on growth, morphology and physiology of plants grown under controlled environment may differ when grown under their more natural settings. Thus, to advance our understanding of how plants respond to elevated CO_2_ under their real-world ecosystem, Free-Air Carbon dioxide Enrichment (FACE) experimental design has been developed. FACE design allows the growth of plants under field condition with artificially controlled elevated CO_2_ concentrations. However, FACE experiment has poor performance than open top chamber experiment because of fluctuating CO_2_ levels. In a FACE experiment, growth of potato plants at elevated CO_2_ enhanced tuber yield and WUE by 34% and 70% respectively in 1998 and, by 53% and 67% respectively in 1999, (Magliulo et al., 2003).

Although the effects of elevated CO_2_ have been studied for many crop species, including potato, there is little information on cultivar specific growth, yield and photosynthetic characteristics of this economically important crop under elevated CO_2_. Additionally, majority of previous studies have focused primarily on photosynthetic parameters and tuber yield with little emphasis on biochemical and molecular analyses. Understanding how potato plants respond to predicted increase in atmospheric CO_2_ at the physiological, biochemical and molecular level is important to improve potato productivity. Thus, the main objectives of the present study are to i) investigate the effects of elevated CO_2_ on the growth, yield and photosynthetic performance and water use efficiency of various commercial potato cultivars, and ii) evaluate tuber yield regulation at the physiological, biochemical and molecular level under elevated CO_2_. In the present study, we measured CO_2_ gas exchange rates and tuber yield in combination with photosynthetic pigments and leaf protein analyses of five distinct potato cultivars under ambient CO_2_ (400 μmol C mol^−1^) versus elevated CO_2_ (750 μmol C mol^−1^) growth conditions.

## Materials and methods

### Plant materials, growth conditions and experimental set-up

Experiments were carried out using five potato (*Solanum tuberosum* L.) cultivars (AC Novachip, Atlantic, Kennebec, Russet Burbank and Shepody) in the growth chambers at Fredericton Research and Development Centre, Fredericton, Canada. Plants were grown in 8^1/4^-inch (4L) clay pots containing a general purpose growing medium with 4 parts soil (Promix BX; Premier Horticulture) and 1 part vermiculite. The plants were grown in the controlled growth chambers (BioChambers TPC-15, Winnipeg, MB, Canada) at either ambient CO_2_ (400 ± 20 μmol CO_2_ mol^−1^) or elevated CO_2_ (750 ± 35 μmol CO_2_ mol^−1^). The plants under both ambient and elevated CO_2_ conditions were grown at 22/16°C day/night temperature regimes, photosynthetic photon flux density (PPFD) of 300 ± 15 μmol photons m^−2^ s^−1^, a 16 h photoperiod and at 50 ± 5% relative humidity. The CO_2_ levels, temperatures, irradiance level, photoperiod and relative humidity in each chamber were computer controlled and, monitored and recorded continuously. The plants were watered everyday including nutrient supplementation every other day. The nutrients were provided by using 20-20-20 NPK fertilizer, and Fe, Mn, Zn, Cu, B, Mo, EDTA supplements (Plant Products Co., Ltd., Brampton, ON, Canada).

In all experiments, one replicate pot for each cultivar in either the ambient or high CO_2_ growth chamber was grown. The position of the pots within each growth chamber was randomly changed twice a week to minimize chamber corner effect. The experiments were repeated three times during 2019–2022 using different growth chambers to minimize growth chamber effects. Thus, all data are the averages of measurements made on three replicate plants from three independent experiments.

Additionally, we conducted an initial experiment to evaluate the potential effects of pot size on rooting volume and sink growth. All five cultivars were grown in pots of varying sizes (6 and 8^1/4^ inches) at either ambient (400 ± 20 µmol CO_2_ mol^−1^) or elevated (750 ± 35 µmol CO_2_ mol^−1^) CO_2_ and under the growth conditions the same as mentioned above. Tubers were harvested from both ambient and elevated CO_2_-grown 140 day-old plants at their maturity and, total tuber weight, tuber size and number were recorded.

### Growth characteristics

Plant height and total leaf number were monitored once a week and data were recorded when they reached maximum height, and had maximum leaf number. Specific leaf fresh weight was calculated as leaf fresh weight in grams per square meter leaf area, and specific leaf dry weight as leaf dry weight in grams per square meter leaf area. Specific leaf weight was determined using a fully expanded physiologically active terminal leaflet (at the 3^rd^ position from the top) of 5-week-old plants grown at either ambient CO_2_ or elevated CO_2_.

### Tuber yield, tuber number and size

Tubers were harvested from both ambient CO_2_ and elevated CO_2_-grown 140 day-old plants at their maturity and, tuber weight and number were recorded. Average tuber size was calculated by dividing total tuber weight with total tuber number for each cultivar under both ambient and elevated growth conditions.

### Physiological and biochemical measurements and analyses

All physiological and biochemical measurements and analyses were subsequently performed on a single fully expanded physiologically active terminal leaflet (at the 3^rd^ position from the top) of 5-week-old plants grown at either ambient CO_2_ or elevated CO_2_ as described in the following headings.

### Measurements of photosynthesis, water use efficiency and stomatal characteristics

CO_2_ gas exchange rates were measured on fully expanded terminal leaflets (at the top 3^rd^ position) by using the LI-COR 6400 portable infrared CO_2_ gas analyzer (LI-6400 XRT Portable Photosynthesis System; LI-COR Biosciences, Lincoln, NE, U.S.A.). Rates of net photosynthesis (*A*) was measured at either growth irradiance (300 PPFD) or saturating irradiance (1600 PPFD), and under respective growth CO_2_ of either 400 μmol CO_2_ mol^−1^ or 750 μmol CO_2_ mol^−1^. Respiration rates were measured in the dark. Gross photosynthesis was calculated as the sum of rates of net photosynthesis and respiration. In addition, stomatal conductance (*g_s_*) and leaf transpiration rates (*T*) were obtained simultaneously with CO_2_ gas exchange measurements. Leaf instantaneous water use efficiency (iWUE) was calculated as the rate of CO_2_ assimilation divided by the rates of transpiration (*A*/*T*).

### Chlorophyll a fluorescence measurements

Room temperature Chl *a* fluorescence was measured concurrently with CO_2_ gas exchange on the fully expanded terminal leaflets using a LI-COR 6400. A standard fluorescence leaf chamber of 2 cm^2^ was used to obtain all measurements of Chl *a* fluorescence. The leaves were dark-adapted for 20 min prior to fluorescence measurements to ensure that all photosystem II (PSII) reaction centers were open. This enabled us to obtain the minimum fluorescence (*F_o_)* and maximal fluorescence (*F_m_)* from the dark-adapted leaf as previously suggested (Maxwell and Johnson, 2000). The leaves were then provided with an actinic light of 300 μmol photons m^−2^ s^−1^ to determine the minimum fluorescence (*F’_o_*), maximal fluorescence (*F’_m_*) and steady-state fluorescence (*F_s_*) from the light-adapted leaves (Maxwell and Johnson, 2000).

The maximal quantum yield of PSII was calculated as *F*_v_/*F*_m_ as previously described (Maxwell and Johnson, 2000). Linear electron transport rates (ETR) through PSII was determined as ETR= (Φ_PSII_)(PPFD)(0.84)(0.5), where Φ_PSII_ represents the operating efficiency of PSII (Baker, 2008). Non-photochemical quenching (NPQ), a measure of heat dissipation of excess light energy, was calculated as: NPQ = (*F*_m_ − *F’*_m_)/*F’*_m_ (Maxwell and Johnson, 2000). Excitation pressure, a measure of closed PSII reaction centres, was determined as 1-qP, where qP represents the photochemical quenching of light energy through PSII.

### Determination of total leaf protein and chlorophyll content

Total leaf protein from both ambient CO_2_ and elevated CO_2_-grown plants was determined as previously described (Gervais et al., 2021) to evaluate the effects of elevated CO_2_ on leaf protein content. Following each CO_2_ gas exchange measurement, the fully expanded physiologically active leaves from both ambient CO_2_ and elevated CO_2_-grown plants were harvested, immediately frozen in liquid N_2_ and stored at −80°C for further analysis. The frozen leaf samples were ground into a fine powder using liquid N_2_ in a mortar and pestle. About 30–35 mg of ground leaf samples were added to 800 μl of cold (4°C) extraction buffer containing 1 M Tris-HCl (pH 6.8), 10% (w/v) SDS, 15% (w/v) sucrose and 0.5 M DTT. The samples were vortexed briefly, solubilized at 70°C for 10 min and centrifuged to remove debris. Total leaf protein concentrations of the supernatant were quantified using a modified Lowry method (Larson et al., 1986). While quantifying the total leaf protein content, the addition of 1 μg of bovine serum albumin (Invitrogen, Carlsbad, CA) in the extraction buffer was used as an internal standard.

For chlorophyll analysis, the leaf samples were ground into a fine powder and total chlorophyll, chlorophyll *a* (Chl *a*), and chlorophyll *b* (Chl *b*) content were estimated according to Arnon (1949).

### Statistical analysis

The experiments were replicated three times. Thus, data for all measurements and biochemical analyses were averages of three replicates from three independent experiments. Statistical analyses were performed using ANOVA in Prism 7.0 (GraphPad Software Inc.). When conducting anova, growth parameters, CO_2_ gas exchange, photosynthetic and fluorescence parameters, leaf protein and pigment content were considered as dependent variables while the CO_2_ levels were considered as independent variables. Means were compared at the 5% level of significance (P ≤ 0.05) between ambient versus elevated CO_2_-grown plants within each cultivar.

## Results

### Effects of pot size on tuber yield, tuber number and size

We conducted an initial experiment to evaluate the potential effects of pot size on rooting volume and sink growth by growing plants in pots of varying sizes (6 and 8^1/4^ inches). At ambient CO_2_, pot size had minimal effects on total tuber weight, tuber size and number (data not shown). However, at elevated CO_2_, although tuber numbers were minimally affected, total tuber weight and tuber size were significantly affected by variations in pot sizes, such that total tuber weight and tuber size increased substantially with larger pot size for all cultivars (data not shown). Thus, to minimize rooting volume constraints and sink limitations, all five cultivars were grown in 8^1/4^ inch-sized pots in all experiments regardless of growth CO_2_.

### Effects of elevated CO_2_ on growth characteristics

Elevated CO_2_ had minimal effects on growth habit and leaf morphology of all cultivars tested relative to their respective controls at ambient CO_2_ (**Table 1**). Elevated CO_2_ slightly increased the plant height for AC Novachip and Shepody but slightly decreased the height for Atlantic, Kennebec and Russet Burbank (**Table 1**). Similarly, total leaf number increased minimally and for AC Novachip and Kennebec but decreased minimally for Atlantic, Russet Burbank and Shepody under elevated versus ambient CO_2_ growth conditions (**Table 1**). Except for Russet Burbank, all other cultivars exhibited a minimum increase in the specific leaf fresh weight (SLW, g fresh weight m^−2^ leaf area) when grown at elevated CO_2_ as compared to at ambient CO_2_ (**Table 1**). Elevated CO_2_ significantly increased the specific leaf dry weight (SLW, g dry weight m^−2^ leaf area) by 20% for Shepody but had minimal effects for other cultivars as compared to at ambient CO_2_ (**Table 1**).

**Table 1:** Effects of elevated CO_2_ on plant morphology and growth characteristics of five potato genotypes grown under ambient CO_2_ (400 μmol CO_2_ mol^−1^) and elevated CO_2_ (750 μmol CO_2_ mol^−1^).

Cultivars	Growth CO_2_	Growth characteristics						
		Plant height (cm)	Total leaf number (plant^-1^)	SLFW (g fresh weight m^-2^ leaf area)	SLDW (g dry weight m^-2^ leaf area)	Total chlorophyll (mg m^-2^ leaf area)	Total protein (g m^-2^ leaf area)	*F_v_/F_m_*
AC Novachip	Ambient	84 ± 13	1284 ± 108	193 ± 7	24 ± 2.6	217 ± 26	19.6 ± 2.2	0.81 ± 0.02
	Elevated	89 ± 7	1398 ± 71	218 ± 16	27 ± 2.9	195 ± 29	17.3 ± 1.9	0.80 ± 0.01
Atlantic	Ambient	80 ± 9	1043 ± 164	225 ± 19	31 ± 3.5	296 ± 15	14.8 ± 1.7	0.80 ± 0.01
	Elevated	74 ± 16	841 ± 49	246 ± 27	33 ± 1.2	223 ± 21*	19.7 ± 1.1*	0.83 ± 0.01
Kennebec	Ambient	91 ± 15	821 ± 136	252 ± 23	33 ± 1.8	303 ± 17	16.6 ± 2.4	0.81 ± 0.02
	Elevated	89 ± 11	942 ± 152	267 ± 35	36 ± 2.4	201 ± 10**	14.1 ± 1.2	0.82 ± 0.03
Russet Burbank	Ambient	112 ± 13	928 ± 76	238 ± 9	27 ± 0.7	262 ± 11	20.3 ± 2.1	0.80 ± 0.01
	Elevated	96 ± 9	785 ± 133	203 ± 18	26 ± 1.8	206 ± 17*	18.5 ± 3.1	0.82 ± 0.01
Shepody	Ambient	81 ± 8	802 ± 69	192 ± 14	25 ± 2.1	274 ± 39	18.7 ± 1.0	0.79 ± 0.02
	Elevated	96 ± 12	781 ± 116	205 ± 32	30 ± 1.3*	245 ± 26	14.1 ± 1.5*	0.83 ± 0.01

Measurements for specific leaf weight, leaf protein content, leaf chlorophyll content, and *F_v_/F_m_* were performed on the fully expanded terminal leaflets of 5- week- old ambient and elevated CO_2_−grown plants. Data represent the averages of three experiments ± SE. Significant differences of the means between ambient CO_2_ versus elevated CO_2_-grown plants within each cultivar are indicated by the symbol * (*P* ≤ 0.05), ** (*P* ≤ 0.01). SLFW, specific leaf fresh weight; SLDW, specific leaf dry weight; *F_v_/F_m_*, photochemical efficiency of photosystem II.

### Effects of elevated CO_2_ on tuber yield, tuber number and size

**Figure 1:**
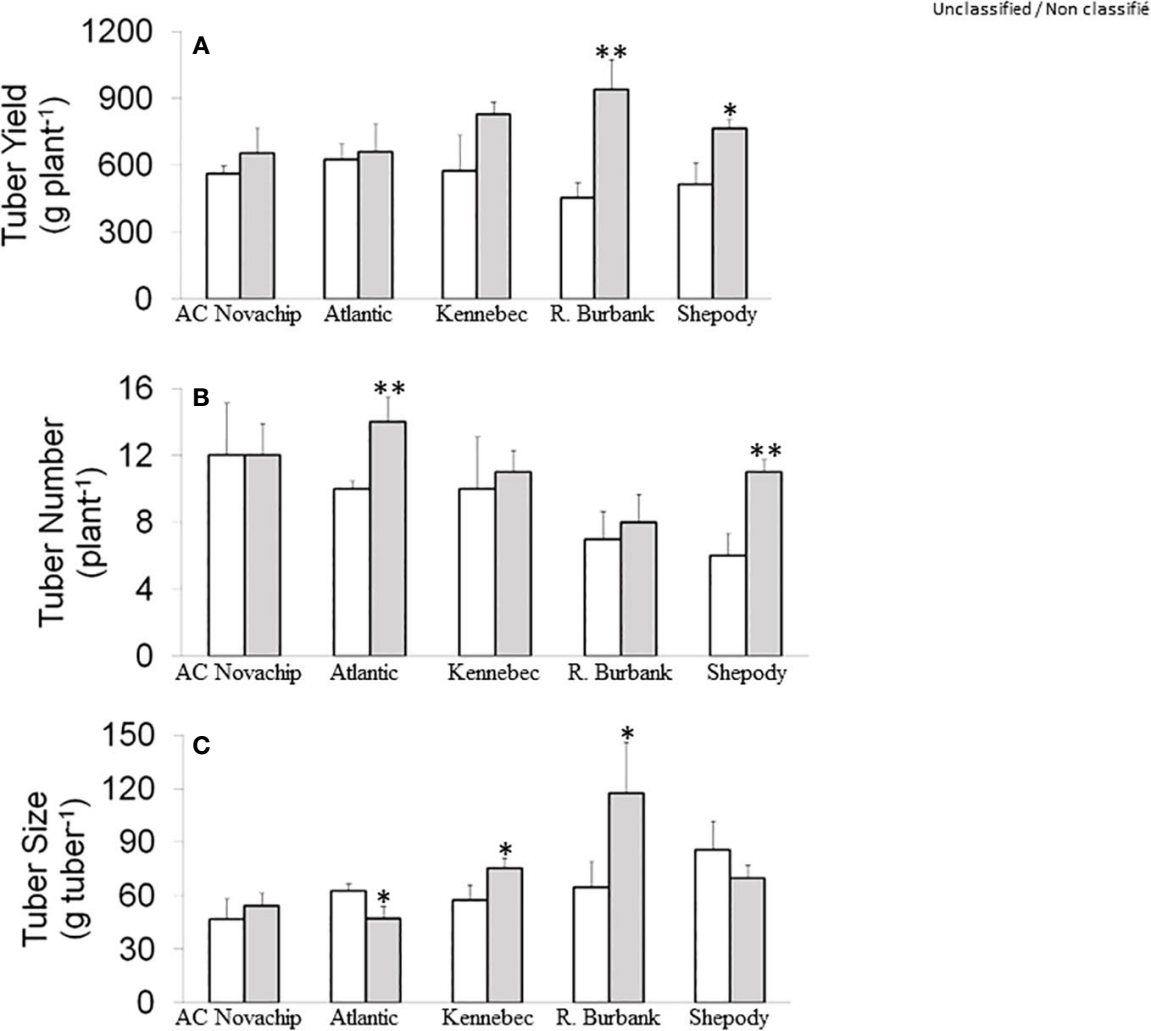
Tuber yield per plant **(A)**, tuber number per plant **(B)** and average tuber size **(C)** of five potato genotypes grown under ambient CO_2_ of 400 μmol CO_2_ mol^−1^ (open bars) and elevated CO_2_ of 750 μmol CO_2_ mol^−1^ (closed bars). Data represent the averages of three experiments ± SE. Significant differences of the means between ambient versus elevated CO_2_-grown plants within each cultivar are indicated by the symbol * (*P* ≤ 0.05), ** (*P* ≤ 0.01). R. Burbank, Russet Burbank.

Under ambient CO_2_, total tuber yield per plant ranged from 454 g to 626 g across the cultivars (**Figure 1A**, open bars). The tuber yield of Russet Burbank and Shepody was comparable when grown at ambient CO_2_ but was slightly lower from those of AC Novachip, Atlantic and Kennebec (**Figure 1A**, open bars). Growth of potato plants at elevated CO_2_ increased total tuber yield for all cultivars compared to their counterparts at ambient CO_2_ (**Figure 1A**, open versus closed bars). However, the CO_2_-stimulation of tuber yield varied across the cultivars such that the stimulation was significantly higher for Russet Burbank (107%) and Shepody (49%) (**Figure 1A**, open versus closed bars). Although we observed an increase in the total tuber yield of 16%, 6% and 44% for AC Novachip, Atlantic and Kennebec respectively at elevated CO_2_, those increments were statistically insignificant as compared to their respective controls at ambient CO_2_ (**Figure 1A**, open versus closed bars).

Under ambient CO_2_, total number of tubers per plant were 6–12 across the cultivars (**Figure 1B**, open bars). Elevated CO_2_ significantly increased the total tuber number for Atlantic (40%) and Shepody (83%) but had minimal effects for AC Novachip (0%), Kennebec (10%) and Russet Burbank (14%) (**Figure 1B**, open versus closed bars). Since we observed varied effects of tuber number on total tuber yield across the cultivars, we figured out whether the increase in tuber yield under elevated CO_2_ was accounted for by the tuber size.

Growth at elevated CO_2_ significantly increased average tuber size by about 30% and 80% for Kennebec and Russet Burbank respectively (**Figure 1C**, open versus closed bars). In contrast, elevated CO_2_ significantly decreased the average tuber size by 25% for Atlantic but had minimal effects for AC Novachip and Shepody (**Figure 1C**, open versus closed bars).

### Effects of elevated CO_2_ on the rates of photosynthesis

**Figure 2:**
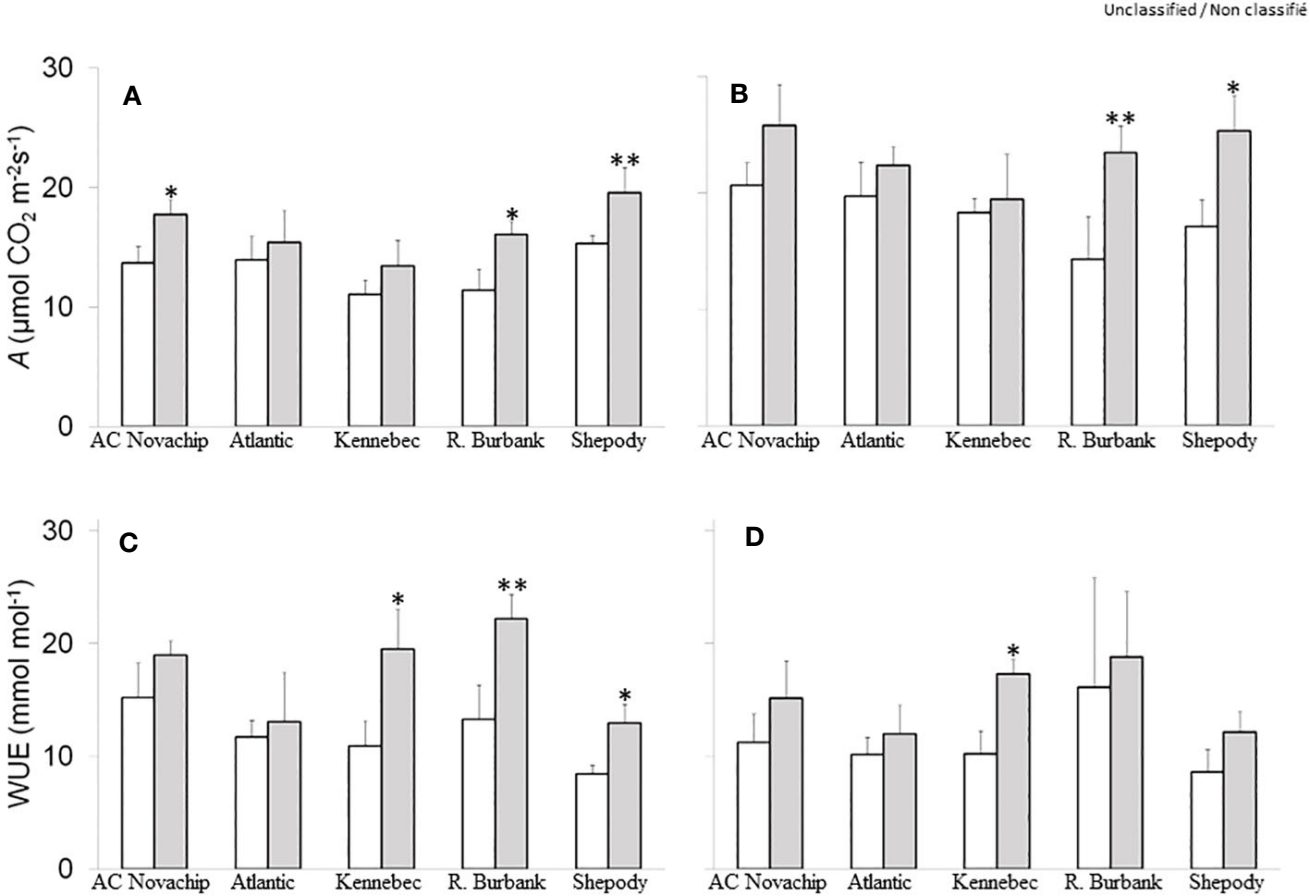
Effects of elevated CO_2_ on rates of gross photosynthesis **(A, B)** and on instantaneous water use efficiency **(C, D)** of five potato genotypes grown under ambient CO_2_ of 400 μmol CO_2_ mol^−1^ (open bars) and elevated CO_2_ of 750 μmol CO_2_ mol^−1^ (closed bars) respectively. Measurements were obtained at either growth irradiance of 300 PPFD **(A, C)** or saturating irradiance of 1600 PPFD **(B, D)**. Data represent the averages of three experiments ± SE. Significant differences of the means between ambient versus elevated CO_2_-grown plants within each cultivar are indicated by the symbol * (*P* ≤ 0.05), ** (*P* ≤ 0.01). A, gross photosynthesis; WUE, instantaneous water use efficiency. R. Burbank, Russet Burbank.

Gas exchange rates were measured to characterize photosynthesis of various potato genotypes under elevated CO_2_. **Figures 2A, B** illustrate the rates of gross CO_2_ assimilation for all five cultivars measured at their respective growth CO_2_ (400 µmol CO_2_ mol^−1^, open bars; 750 µmol CO_2_ mol^−1^, closed bars), and at either growth irradiance (300 PPFD, **Figure 2A**) or at saturating irradiance (1600 PPFD, **Figure 2B**). When grown at ambient CO_2_ and measured at growth irradiance, all cultivars exhibited a comparable gross photosynthetic rates of 11–15 µmol CO_2_ m^−2^ s^−1^ (**Figure 2A**, open bars). Growth at elevated CO_2_ significantly stimulated the rates of gross photosynthesis by 30%, 41% and 28% for AC Novachip, Russet Burbank and Shepody respectively as compared to their ambient CO_2_-grown counterparts when measured at growth irradiance (**Figure 2A**; open versus closed bars). Growth at elevated CO_2_ had insignificant effects on the rates of gross photosynthesis for Atlantic and Kennebec (**Figure 2A**; open versus closed bars). As expected, measuring at saturating irradiance of 1600 PPFD increased the rates of gross photosynthesis for all cultivars grown at either ambient (**Figure 2A** versus **2B**, open bars) or elevated CO_2_ (**Figure 2A** versus **2B**, closed bars) as compared to those rates obtained when measured at growth irradiance of 300 PPFD. Similar to at 300 PPFD measuring irradiance, all cultivars exhibited CO_2_-stimulation of rates of gross photosynthesis when measured at saturating irradiance of 1600 PPFD. In fact, the CO_2_-stimulation of rates of gross photosynthesis was magnified for Russet Burbank and Shepody when measured at saturating irradiance relative to at growth irradiance. At saturating light, Russet Burabank and Shepody exhibited a significant increase of 64% and 48% of rates of gross photosynthesis respectively at elevated CO_2_ relative to at ambient CO_2_ (**Figure 2B**, open versus closed bars). The CO_2_-stimulation of rates of gross photosynthesis was 25%, 14% and 6% for AC Novachip, Atlantic and Kennebec respectively, however these stimulations were statistically insignificant (**Figure 2B**; open versus closed bars).

### Effects of elevated CO_2_ on instantaneous water use efficiency and stomatal characteristics

Under ambient CO_2_, we observed instantaneous water use efficiency (iWUE) of about 8–15 mmol mol^−1^ at 300 PPFD measuring irradiance across the cultivars (**Figure 2C**, open bars). Growth at elevated CO_2_ differentially enhanced iWUE for all cultivars by about 11% to 79% although this enhancement was significant for Kennebec, Russet Burbank and Shepody only at 300 PPFD (**Figure 2C**, open versus closed bars). Similar responses of CO_2_-enhancement of iWUE were observed when measured at 1600 PPFD except that the enhancement was significant for Kennebec only (**Figure 2D**, open versus closed bars).

**Figure 3:**
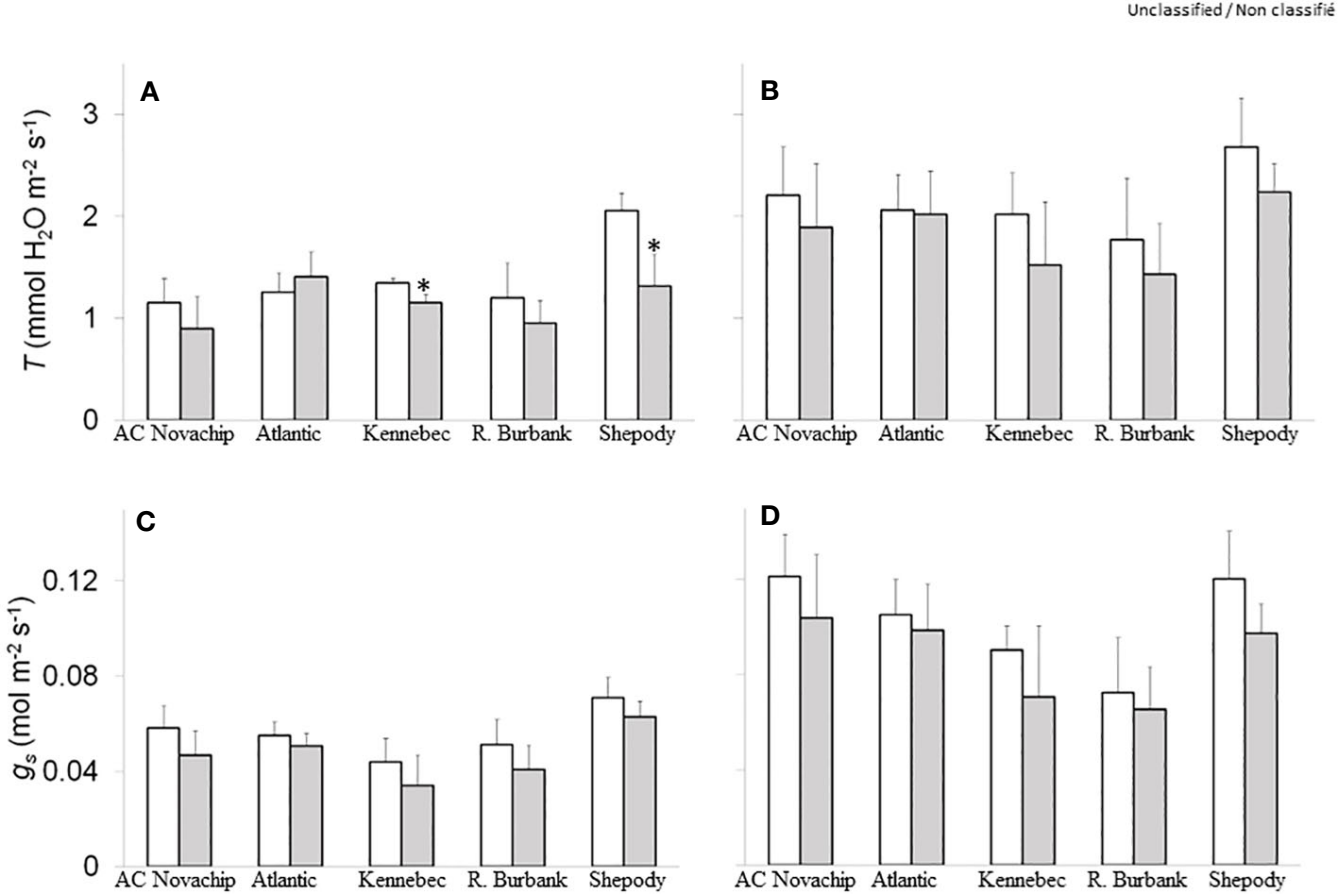
Rates of transpiration **(A, B)** and stomatal conductance **(C, D)** of five potato genotypes grown under ambient CO_2_ of 400 μmol CO_2_ mol^−1^ (open bars) and elevated CO_2_ of 750 μmol CO_2_ mol^−1^ (closed bars). Measurements were obtained at either growth irradiance of 300 PPFD **(A, C)** or saturating irradiance of 1600 PPFD **(B, D)**. Data represent the averages of three experiments ± SE. Significant differences of the means between ambient versus elevated CO_2_-grown plants within each cultivar are indicated by the symbol * (*P* ≤ 0.05). *T*, transpiration; *g_s_*, stomatal conductance. R. Burbank, Russet Burbank.

Transpiration rates varied across the cultivars irrespective of measuring irradiance at ambient CO_2_ (**Figures 3A, B**, open bars). Elevated CO_2_ inhibited transpiration rates by about 22%, 14%, 21% and 36% for AC Novachip, Kennebec, Russet Burbank and Shepody respectively when measured at growth irradiance although the inhibition was only significant for Kennebec and Shepody (**Figure 3A**, open versus closed bars). In contrast, elevated CO_2_ increased the transpiration rates by 12% for Atlantic when measured at growth irradiance (**Figure 3A**, open versus closed bars). When measured at saturating irradiance, although we observed a general trend of decreased transpiration rates for all cultivars at elevated CO_2_ compared to their respective ambient controls, these differences were statistically not significant (**Figure 3B**, open versus closed bars).

Consistent with the transpiration rates, we observed differential rates of stomatal conductance across the cultivars irrespective of measuring irradiance at ambient CO_2_ (**Figures 3C, D**, open bars). Elevated CO_2_ minimally suppressed the stomata conductance by about 8–22% for all cultivars as compared to ambient controls at 300 PPFD (**Figure 3C**, open versus closed bars). Similar responses were observed at 1600 PPFD measuring irradiance (**Figure 3D**, open versus closed bars).

### Effects of elevated CO_2_ on fluorescence parameters

*In vivo* Chl *a* fluorescence was monitored simultaneously with the CO_2_ gas exchange to estimate i) the maximum photochemical efficiency of photosystem II (PSII) in the dark-adapted state (*F_v_/F_m_*), ii) the photosynthetic electron transport rates through PSII (ETR), iii) non-photochemical quenching (NPQ), the capacity to dissipate excess energy as heat and, (iv) excitation pressure (1-qP), a measure of closed PSII reaction centres.

**Figure 4:**
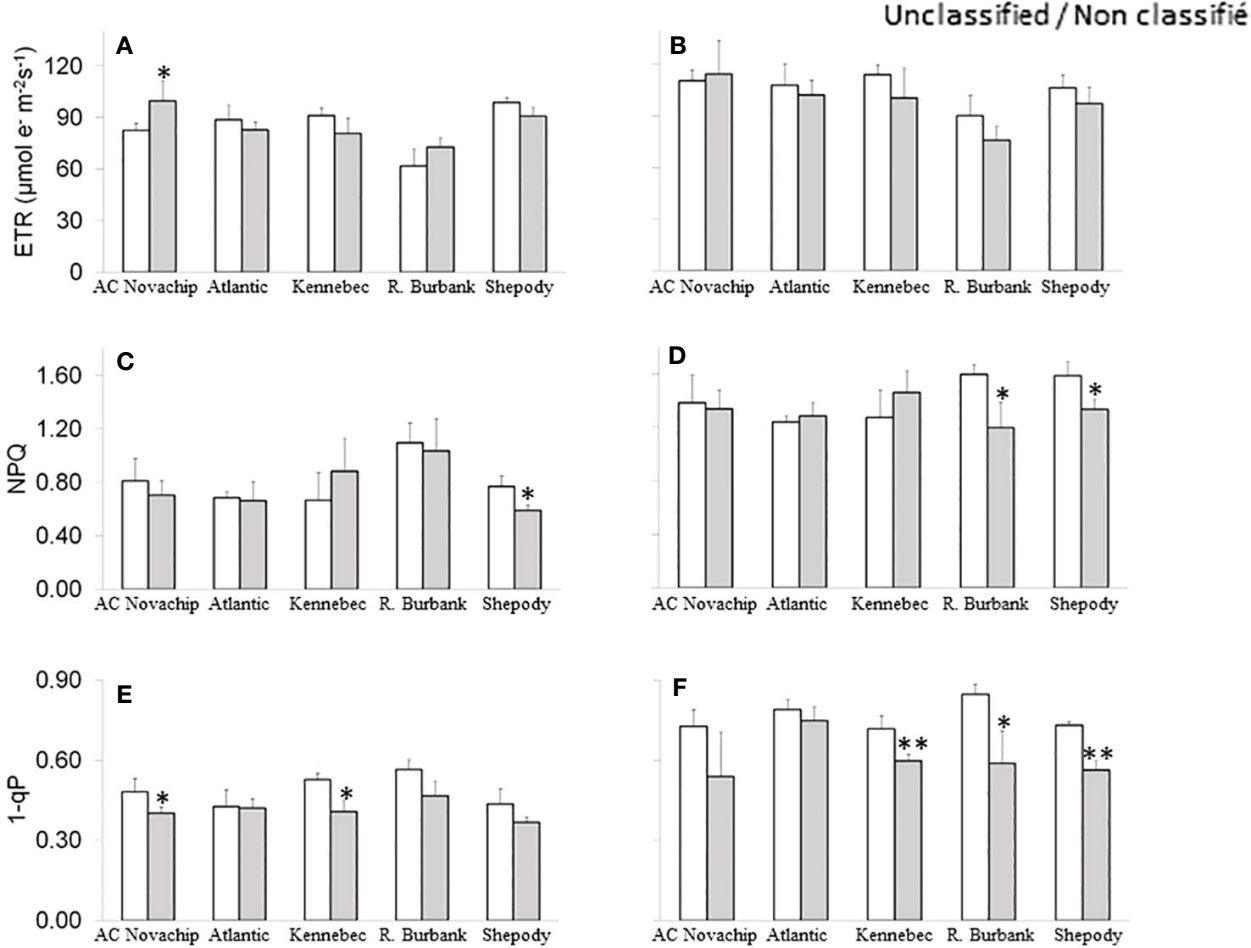
Effects of elevated CO_2_ on electron transport rates **(A, B)**, non-photochemical quenching **(C, D)** and excitation pressure **(E, F)** of five potato genotypes grown under ambient CO_2_ of 400 μmol CO_2_ mol^−1^ (open bars) and elevated CO_2_ of 750 μmol CO_2_ mol^−1^ (closed bars). Measurements were obtained at either growth irradiance of 300 PPFD **(A, C, E)** or saturating irradiance of 1600 PPFD **(B, D, F)**. Data represent the averages of three experiments ± SE. Significant differences of the means between ambient versus elevated CO_2_-grown plants within each cultivar are indicated by the symbol * (*P* ≤ 0.05), ** (*P* ≤ 0.01). ETR, electron transport rates; NPQ, non-photochemical quenching; 1-qP, excitation pressure. R. Burbank, Russet Burbank.

We observed a comparable maximum photochemical efficiency of PSII in the dark-adapted state (*F_v_/F_m_*) across the cultivars either at ambient or elevated CO_2_ (**Table 1**). Additionally, there were minimal changes on *F_v_/F_m_* for all cultivars upon growth at elevated CO_2_ as compared to those observed for their ambient-CO_2_ controls (**Table 1**). The electron transport rates (ETR) increased for all cultivars measured at saturating irradiance compared to those rates observed when measured at growth irradiance regardless of growth CO_2_ (**Figure 4A** versus **4B**). Elevated CO_2_ significantly enhanced ETR for AC Novachip when measured at growth irradiance (**Figure 4A**, open versus closed bar) but had minimal effects for other cultivars at either growth irradiance (**Figure 4A**, open versus closed bars) or saturating irradiance (**Figure 4B**, open versus closed bars).

At ambient CO_2_, NPQ varied from 0.66 to 1.09 across the cultivars when measured at growth irradiance (**Figure 4C**, open bars). Measuring at saturating irradiance considerably increased these values and ranged from 1.24 to 1.59 across the cultivars at ambient CO_2_ (**Figure 4D**, open bars). Elevated CO_2_ significantly decreased NPQ for Shepody but had minimal effects on other cultivars when measured at growth irradiance (**Figure 4C**, open versus closed bars). At saturating light, we observed significant decrease in NPQ for Russet Burbank in addition to Shepody under elevated CO_2_ conditions as compared to their counterparts at ambient CO_2_ (**Figure 4D**, open versus closed bars).

As expected, measuring at saturating irradiance substantially increased excitation pressure for all cultivars relative to when measured at growth irradiance at either ambient CO_2_ (**Figure 4E** versus **4F**, open bars) or elevated CO_2_ (**Figure 4E** versus **4F**, closed bars). Although elevated CO_2_ inhibited excitation pressure for all cultivars by up to 23% as compared to their ambient controls, the inhibition was only significant for AC Novachip and Kennebec when measured at growth irradiance (**Figure 4E**, open versus closed bars). When measured at saturating irradiance, elevated CO_2_ significantly suppressed excitation pressure by 17%, 31% and 23% for Kennebec, Russet Burbank and Shepody respectively but had minimal effects for AC Novachip and Atlantic (**Figure 4F**, open versus closed bars).

### Effects of elevated CO_2_ on leaf protein and chlorophyll content

Growth at elevated CO_2_ significantly increased the total leaf protein content by 33% for Atlantic but significantly decreased by 25% for Shepody as compared to their ambient controls (**Table 1**). Minimal differences in the total leaf protein content were observed for AC Novachip, Kennebec and Russet Burbank under elevated versus ambient CO_2_ growth conditions (**Table 1**).

We observed a trend of lower leaf chlorophyll content per unit leaf area for all cultivars upon growth and development at elevated CO_2_ compared to at ambient CO_2_. However, these reductions were only significant and decreased by 25%, 34% and 21% for Atlantic, Kennebec and Russet Burbank respectively (**Table 1**).

## Discussion

There is an utmost need to double the current yield of major food crops to secure the nutritional requirements of the rising global population over the next 50 years (Murchie et al., 2009). Such increasing food demand comes at a time when the atmospheric CO_2_ concentration is expected to continue to rise in the future. Thus, it is crucial to identify specific crop species, cultivars and characteristics to develop and exploit new strategies aim at enhancing yield potentiality under predicted future CO_2_ environment. As a fourth major and staple food crop, potato could be a candidate food crop to fulfill the increased nutritional demand and keep pace with the growing population worldwide (FAOSTAT, 2021). Understanding how potato plants senses and responds to future increase in atmospheric CO_2_ at the physiological, biochemical and molecular level is therefore, important to maintain viable potato industries globally. The effects of long-term growth and development at elevated CO_2_ on photosynthesis, respiration and biomass accumulation have been extensively studied in several plant species for decades, but experimental results have varied widely. Most of the studies reveal that elevated CO_2_ enhances tuber yield and rates of photosynthesis for potatoes, however there is limited in-depth studies on effects of elevated CO_2_ across different commercial potato cultivars. In this study we asked the question, whether CO_2_-stimulation of tuber yield and photosynthetic characteristics varies across potato genotypes using five commercial cultivars.

Consistent with previous studies (Högy and Fangmeier, 2009; Wheeler et al., 2019; Chen and Setter, 2021), growth at elevated CO_2_ substantially increased total tuber yield for Russet Burbank and Shepody (**Figure 1A**, open versus closed bars). However, elevated CO_2_ had minimal effects on total tuber yield for AC Novachip, Atlantic and Kennebec. We recorded total tuber number to assess whether the differential increase in total tuber yield across potato cultivars under elevated CO_2_ was associated with variations in tuber number. Although growth at elevated CO_2_ increased total tuber number significantly for Atlantic and Shepody, elevated CO_2_ had minimal effects on tuber number for AC Novachip, Kennebec and Russet Burbank (**Figure 1B**, open versus closed bars). In contrast, elevated CO_2_ significantly increased the average tuber size for Russet Burbank but minimally decreased the size for Shepody (**Figure 1C**, open versus closed bars). This suggests that the CO_2_-enhancement of tuber yield for Shepody and Russet Burbank was due in part to increased tuber number and size respectively under elevated CO_2_. Lee et al. (2020) revealed that the increase in tuber yield under elevated CO_2_ was mainly accounted for by larger mean tuber size rather than by tuber number. Similarly, elevated CO_2_ enhanced tuber yield by 40% at either at 550 or 680 µmol CO_2_ mol^−1^ owing to increase in tuber size with tuber number having minimal effect (Donnelly et al., 2001). In our study, when taken all cultivars together, we conclude that the variations in stimulation of tuber yield across cultivars under elevated CO_2_ was associated with the differences in increase in tuber number and size or combination of both, depending on the cultivars, under elevated CO_2_.

During photosynthesis, CO_2_ is assimilated to carbohydrates using ATP and NADPH through Calvin-Benson cycle in the source leaves (Stitt et al., 2010; Rochaix, 2011; Foyer et al., 2012; Paul, 2013; Sharkey, 2021). The photosynthetic end product, sucrose, is subsequently translocated via phloem loading into the underground stem where it is converted to starch, which then accumulates in the stolon giving rise to tuber formation (Dahal et al., 2019). It is therefore, the tuber yield, number and size of potato crop are determined primarily by an effective coordination of these processes between the source (photosynthesis) and sink (tuber). Since carbohydrate is the major component of potato tubers, we asked the questions whether CO_2_-stimulation of rates of photosynthesis contributed to increased tuber yield under elevated CO_2_. The increased in tuber yield for Russet Burbank and Shepody (**Figure 1A**, open versus closed bars) was consistent with significant increase in the rates of photosynthesis under elevated CO_2_ (**Figure 2A**, open versus closed bars). In contrast, although we observed a significant increase in the rates of photosynthesis for AC Novachip under elevated CO_2_ (**Figure 2A**, open versus closed bars.), the CO_2_-stimulation of tuber yield was not significantly different (**Figure 1A**, open versus closed bars). The minimal increase in the rates of photosynthesis for Atlantic and Kennebec under elevated CO_2_ was at par with minimal increase in the tuber yield. Taken together, we can conclude that the increase in the rates of photosynthesis under elevated CO_2_ does contribute, in part to the enhanced tuber yield under elevated CO_2_. This is consistent with previous study by Wheeler et al. (2019), who revealed that the increase in tuber yield under elevated CO_2_ was associated with the CO_2_-stimulation of rates of photosynthesis. Similarly, Yubi et al. (2021) reported an increase in the rates of photosynthesis, which was reflected in to higher tuber yield under elevated CO_2_. The CO_2_-stimulation of tuber yield was in fact, more pronounced when combined with higher growth temperature (Yubi et al., 2021). The results obtained for AC Novachip suggests that the other phenomenon such as the source–sink relationship can change under elevated CO_2_ (Fleisher et al., 2008; Lee et al., 2020). Additionally, the translocation of sucrose from source leaves to underground stem and the conversion of sucrose to starch in the stolon could play a regulating role to determine tuber yield under elevated CO_2_ (Dahal et al., 2019). So, the further study needs to evaluate the effects of elevated CO_2_ on carbon partitioning to different tissues, carbon translocation to stolon and starch synthesis.

One of the important physiological response of the plants to elevated CO_2_ is an increase in water use efficiency (Leakey et al., 2009; Cao et al., 2022). The cultivars Kennebec, Russet Burbank and Shepody exhibited a substantial increase in iWUE than did AC Novachip and Atlantic under elevated CO_2_ at growth irradiance (**Figure 2C**, open versus closed bars). This was consistent with significant increase in the rates of photosynthesis for Russet Burbank and Shepody, and minimal changes for remaining other cultivars under elevated CO_2_ at growth irradiance (**Figure 2A**, open versus closed bars). In contrast to the rates of photosynthesis, except for Kennebec and Shepody, elevated CO_2_ had minimal effects on transpiration rates for all other cultivars at growth irradiance (**Figure 3A**, open versus closed bars). Additionally, there were only minimal changes in stomatal conductance between elevated versus ambient CO_2_-grown plants for all five cultivars (**Figure 3C**, open versus closed bars). This suggests that the increased iWUE of potato cultivars under elevated CO_2_ was contributed mainly by enhanced rates of photosynthesis while transpiration rates and stomatal conductance having varying impact under elevated CO_2_. Previous studies have suggested that higher water use efficiency of potato plants grown under elevated CO_2_ was accounted for by decreased stomatal conductance and lower transpiration rates in addition to increased rates of photosynthesis (Fleisher et al., 2008; Kaminski et al., 2014). However, our current study clearly reveals that the differential stimulation of iWUE of potato cultivars under elevated CO_2_ was mainly accounted for by the differential stimulation of rates of photosynthesis under elevated CO_2_ (**Figure 2A**, open versus closed bars) while stomatal conductance and transpiration rate had minimal effects.

*In vivo* Chlorophyll a (Chl *a*) fluorescence measurements have been extensively used to evaluate the PSII photochemistry and structure, photosynthetic efficiency and overall plant performance under various growth conditions (Baker, 2008). Chl *a* fluorescence is a useful technique to non-destructively monitor the flux of light energy absorbed through photosynthetic pigments. For instance, with this technique researchers can estimate the partitioning of absorbed light energy towards i) PSII photochemistry, where the energy is used to initiate ETR generating ATP and NADPH that are eventually utilized in carbon assimilation, or ii) non-photochemical quenching initiated thermal dissipation mechanisms of excess energy (Baker, 2008). The light energy that is not utilized by PSII photochemistry can also be measured as proportion of closed PSII reaction centres (1-qP) known as excitation energy (Huner et al., 1998).

Although growth at elevated CO_2_ significantly increased the rates of photosynthesis for AC Novachip, Russet Burbank and Shepody relative to their ambient controls (**Figure 2A**, open versus closed bars), we observed a significant increase in ETR for AC Novachip only under elevated CO_2_ as compared to at ambient CO_2_ (**Figure 4A**, open versus closed bars). As described earlier, ETR generates energy in the form of ATP and NADPH, which are then consumed by Calvin-Benson cycle to assimilate CO_2_. Our expectation was that the growth at elevated CO_2_ should increase ETR to meet the ATP and NADPH demand for enhanced carbon assimilation under elevated CO_2_. In fact, for Atlantic and Kennebec, although elevated CO_2_ minimally increased the rates of photosynthesis, it minimally decreased ETR as compared to at ambient CO_2_. It could be possible that the ATP and NADPH generated through ETR may have been diverted to carboxylation reaction from that of photorespiratory oxygenation reaction of Rubisco. We suggest that enhanced carbon assimilation at elevated CO_2_ was due in part to suppressed photorespiration as more CO_2_ relative to O_2_ is available for Rubisco carboxylation reactions. However, this needs to be confirmed in the future research. Since growth at elevated CO_2_ stimulated rates of photosynthesis particularly for AC Novachip, Russet Burbank and Shepody relative to their ambient controls, and had minimal effects on ETR, we should be expecting less NPQ under elevated CO_2_ for these cultivars. However, we observed a significant inhibition in NPQ for Shepody only at elevated CO_2_ as compared to at ambient CO_2_ (**Figure 4C**, open versus closed bars). Although excitation pressure (1-qP) was reduced for all cultivars upon growth at elevated CO_2_ relative to their counterparts at ambient CO_2,_ these reductions were only significant for AC Novachip and Kennebec (**Figure 4E**, open versus closed bars). These all further confirm that the enhanced rates of photosynthesis under elevated CO_2_ was mainly accounted for by increased Rubisco carboxylation and decreased photorespiration with minimal effects of elevated CO_2_ on ETR, NPQ and 1-qP.

Although the effects of elevated CO_2_ on crop yield, photosynthetic performance and water use efficiency have been studied for many crop species, including potato, there is little information on cultivar specific growth and yield of this economically important crop under elevated CO_2_. Elucidating how various potato cultivars respond to predicted increase in atmospheric CO_2_ at the physiological, biochemical and molecular level is important to identify CO_2_-governed key traits that can be used to breed high yielding varieties under future CO_2_ scenario. Taken together, our results suggest a considerable differences in photosynthetic performance, water use efficiency, tuber yield, leaf protein and pigment content among cultivars under elevated CO_2_. We suggest that, based on the target traits, breeder may include specific cultivar in their breeding programs to breed new cultivars that can outperform under rising atmospheric CO_2_. We further suggest that the future study needs to be concentrated on carbon metabolism, carbon translocation, and photosynthetic protein and enzyme activities to fully understand the CO_2_ regulation of photosynthetic performance and tuber yield of potatoes.

## Data availability statement

The original contributions presented in the study are included in the article/supplementary material. Further inquiries can be directed to the corresponding author.

## Author contributions

KD: Writing – original draft, Writing – review & editing. MM: Methodology, Writing – review & editing. TG: Methodology, Writing – review & editing.
